# Characteristics and patient-reported outcomes associated with dropout in severely affected oncological patients: an exploratory study

**DOI:** 10.1186/s12874-021-01259-0

**Published:** 2021-04-20

**Authors:** Pimrapat Gebert, Daniel Schindel, Johann Frick, Liane Schenk, Ulrike Grittner

**Affiliations:** 1 Berlin Institute of Health at Charité –Universitätsmedizin Berlin, Charitéplatz 1, 10117 Berlin, Germany; 2 Charité – Universitätsmedizin Berlin, corporate member of Freie Universität Berlin and Humboldt-Universität zu Berlin, Institute of Biometry and Clinical Epidemiology, Charitéplatz 1, 10117 Berlin, Germany; 3 Charité – Universitätsmedizin Berlin, corporate member of Freie Universität Berlin and Humboldt-Universität zu Berlin, Institute of Medical Sociology and Rehabilitation Science, Charitéplatz 1, 10117 Berlin, Germany

**Keywords:** Cancer, Attrition, Monotone missing data, Non-compliance, Patient-reported outcome measures, Health-related quality of life, Advanced cancer

## Abstract

**Background:**

Patient-reported outcome measures (PROMs) are commonly-used surrogates for clinical outcomes in cancer research. When researching severe diseases such as cancer, it is difficult to avoid the problem of incomplete questionnaires from drop-outs or missing data from patients who pass away during the observation period. The aim of this exploratory study was to explore patient characteristics and the patient-reported outcomes associated with the time-to-dropout.

**Methods:**

In an Oncological Social Care Project (OSCAR) study, the condition of the participants was assessed four times within 12 months (t0: baseline, t1: 3 months, t2: 6 months, and t3: 12 months) by validated PROMs. We performed competing-risk regressions based on Fine and Gray’s proportional sub-distribution hazards model for exploring factors associated with time-to-dropout. Death was considered a competing risk.

**Results:**

Three hundred sixty-two participants were analyzed in the study. 193 (53.3%) completed a follow-up after 12 months, 67 (18.5%) patients dropped out, and 102 patients (28.2%) died during the study period. Poor subjective social support was related to a higher risk of drop-out (SHR = 2.10; 95%CI: 1.01–4.35). Lower values in health-related quality of life were related to drop-out and death. The sub-scales global health status/QoL, role functioning, physical functioning, and fatigue symptom in the EORTC QLQ-C30 were key characteristics of early drop-out.

**Conclusion:**

Severely affected cancer patients with poor social support and poor quality of life seem more likely to drop out of studies than patients with higher levels of social support and a better quality of life. This should be considered when planning studies to assess advanced cancer patients. Methods of close continued monitoring should be actively used when patient experiences a substantial deterioration in their health-related quality of life and symptoms during the study. Results for such studies have to be interpreted with caution in light of specific drop-out mechanisms.

**Trial registration:**

OSCAR study was registered to the German Clinical Trials Register (DRKS-ID: DRKS00013640). Registered 29 December 2017.

**Supplementary Information:**

The online version contains supplementary material available at 10.1186/s12874-021-01259-0.

## Background

Patient-reported outcome measures (PROMs) are tools for assessing a patient’s physical and emotional wellbeing, satisfaction with care, symptoms, and quality of life (QoL) [1]. Patient-reported outcomes (PROs) are usually measured through questionnaires which combine several items into sub-scales or total scales. Oncology research often focuses on PROs as primary outcomes [2], and repeatedly measured PROs are typically observed for exploring and monitoring the change in the health status of cancer patients [3, 4]. As cancer patients are often severely affected by the disease, missing data from drop-outs due to deterioration in health or death are common [5].

Drop-out occurs in a longitudinal study when a participant discontinues the study completely. Rates of drop-out vary from 30 to 50% [6–8] in oncology studies, but reasons for dropping out are not usually recorded [7]. In cancer research, numerous factors have been identified as being related to dropping out. Being older, male, unmarried, having a low level of education and a low economic status are all associated with early dropout. However, the relevance of some of these factors is less consistent than others: some studies show that women are more likely to drop out, for example [6, 9]. Generally, symptom burdens and health conditions are the main factors related to discontinuing a study [6, 7, 10].

Higher rates of drop-out not only result in reduced statistical power, but also cause biased results if subpopulations are over or under-represented in the remaining sample [10]. Knowledge of a patient’s characteristics related to the risk of drop-out will allow for the application of strategies for the minimization of data loss, such as continuous monitoring, reminders, and the use of modern technology (e.g. mobile apps and online questionnaires) to measure data [11] will result in it being more complete and reliable, as is the case with estimation procedures for missing data.

Investigating patient drop-out characteristics in cancer research is useful for study planning with regard to the estimation of sample size, more appropriate definitions of patient inclusion criteria, decreasing insufficient enrollment through improved inclusion criteria, increasing patient retention over study periods, improved monitoring and possible post-recruitment, and purposing appropriate statistical analysis for challenging missing data. The aim of this exploratory study was to assess patient characteristics and the patient-reported outcomes associated with time-to-dropout when accounting for death as a competing risk.

## Methods

### Study population

A protocol for the Oncological Social Care Project (OSCAR) has been reported previously [12]. In short, the OSCAR was developed as an intervention in oncological care by the German company health insurance fund Pronova BKK. A non-randomized, controlled, multi-center intervention study was conducted at three study sites in Germany from January 2018 to February 2020. Three hundred sixty-two participants above the age of 18 with different cancer types were included (see the inclusion criteria in the original protocol [12]). The study recruited severely affected oncological patients with advanced cancer stages, such as metastasized colorectal cancer, malignant neoplasm of the pancreas, lymphoma, or multiple myeloma and malignant plasma cell neoplasms. These patients had a high symptom burden and needed intensive supportive care. One hundred fifty patients in the intervention group and 212 patients in the control group were studied. Patients answered a monthly health-related QoL questionnaire using the European Organization for Research and Treatment of Cancer’s Quality of Life Questionnaire (EORTC QLQ-C30) in the intervention group. The EORTC QLQ-C30 questionnaire for the control group and other PROMs for both groups were assessed at baseline (t0), 3 months (t1), 6 months (t2), and 12 months (t3). Refusal to participate was documented for each follow-up visit, and those who dropped out were asked whether they wanted to discontinue the study because of their health or for other reasons. In the following analysis, we will focus on drop-out (health-related or other reasons) and death.

### Data collection

Demographic data was collected at baseline, e.g. age, sex, time since diagnosis, cancer diagnosis, family status, and education. Education and professional qualifications were classified based on the Comparative Analysis of Social Mobility in Industrial Nations (CASMIN) classification of education [13]. Assessment of subjective social support was based on the Oslo Three-Item Social Support Scale (OSSS-3) [14], where the total score ranges from 3 to 14 points, and is classified as poor (3 to 8), moderate (9 to 11), or strong (12 to 14).

The following five PROMs were assessed in this study:

- The EORTC QLQ-C30 (version 3.0) is a generic tool for assessing the quality of life (QoL) for various cancer patients, and is provided by the European Organisation for Research and Treatment of Cancer [15]. It consists of 30 questions and incorporates nine multi-item scales: five functional scales (physical, role, cognitive, emotional, and social functioning); three symptom scales (fatigue, pain, and nausea/vomiting); six single item scales (dyspnea, insomnia, appetite loss, constipation, diarrhea, and financial difficulties); and a global health status/QoL scale. The total score is calculated by averaging items within scales and transforming them from 0 to 100. Higher values of a functional scale represent a high or healthy level of functioning, while higher values of a symptom scale or item represent a high level of symptomatology or problems.
- The Patient Reaction Assessment (PRA-D) [16] is an instrument used for assessing the perceived quality of the doctor-patient relationship. In OSCAR, we modified five questions using five-point instead of seven-point Likert scales. The total score was transformed using the formula (y = 1.5 * x - 0.5) [17], and ranges from 5 to 35. Higher values indicate better doctor-patient relationships.
- The modified German version of the Autonomy Preference Index (API-DM) [18] consists of two preferences: decision making and information seeking. The decision making preferences consist of four items, and information seeking preferences consist of seven. The total score for each preference is transformed to achieve scores ranging from 0 to 100. Higher values indicate a greater preference for decision making and information seeking.
- The Decision Conflict Scale (DCS) [19] is a self-reported questionnaire for evaluating decision conflicts and comprises ten items, with the sum score ranging from 0 to 100. Higher values indicate greater decision conflicts.
- The European Health Literacy Survey (HLS-EU-Q6) [20] is a shortened, six-item version of the European Health Literacy Survey (HLS-EU) for assessing health literacy (HL). The sum score is averaged and grouped into insufficient (≤2 scores), problematic (2–3 scores), and sufficient (≥3 scores) health literacy.

### Statistical analysis

The study outcome was discontinuation of the study due to either dropout or death. Baseline characteristics were presented separately by participants who completed the study or who dropped out. For continuous variables, mean and standard deviation (SD) and median and interquartile ranges (IQR) are presented, depending on their distribution. For categorical data, absolute and relative frequencies were reported.

Demographic data at baseline, the PROMs at baseline and at the visit before dropping out or death were used for exploring the association with time-to-dropout. Time-to-event was defined from the enrollment date to date of death, date of drop-out, or censored at 12 months. Date of drop-out was defined as when the participant refused to continue the study or the first date of unsuccessful contact try, when the participant could not be reached after trying to contact them three times. Fine and Gray’s proportional sub-distribution hazards models were performed, assuming that death was a competing risk. Cox regression models were used for assessing the association between participant characteristics and death. Multinomial logistic regression models were used for comparing characteristics related to drop-out or death as a sensitivity analysis.

Statistical testing was done within an exploratory framework at a two-sided significance level of α = 0.05 without adjustment for multiple testing. Due to multi-collinearity within PROs (such as a high correlation within the EORTC QLQ-C30 between global health status/QoL and subscales of functional or symptoms) and PROs between baseline and visit prior to drop-out, restricted by the number of observations in some variables (such as family status and HLS-EU-Q6), we only used bivariate models. All the statistical tests were performed using Stata IC15 (StataCorp, 2017, College Station, TX, USA).

## Results

### Participant characteristics and dropout rate

**Fig. 1:**
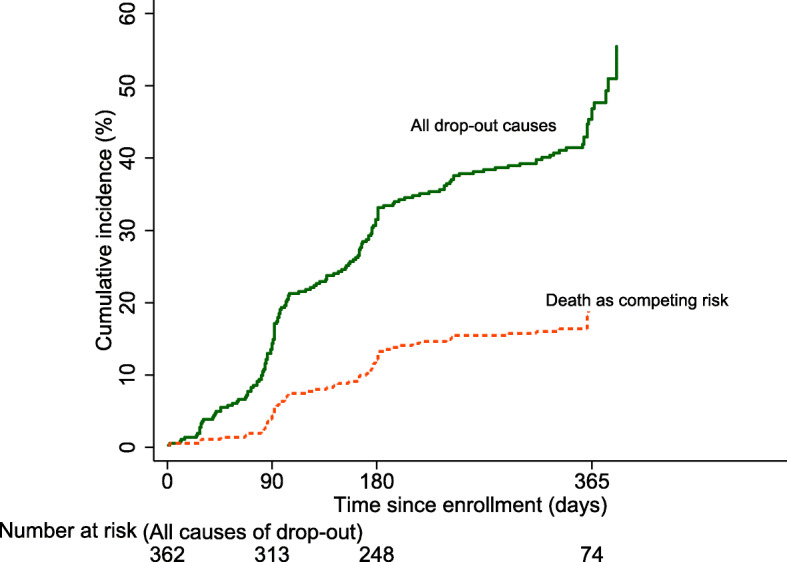
Cumulative incidence of all drop-out causes (green solid line) and drop-out with death as a competing risk (orange dotted line)

Three hundred sixty-two participants were analyzed in the study. One hundred ninety-three patients (53.3%) completed a follow-up at 12 months, 102 died during follow-up (28.2%), and 67 dropped out the study (18.5%) (Additional file 1: Table S1). Rates of drop-out and death combined were 14.4% at 3 months, 31.5% at 6 months, and 46.8% at 12 months (Fig. 1). The participants who dropped out or died were older than the participants who completed the study. However, there was almost no age difference between the participants who dropped out and those who died. The proportion of patients who died was higher in participants with malignant neoplasm of the bronchus and lungs (22.6%) and malignant neoplasm of the pancreas (15.7%) when compared to participants who dropped out and compliant participants (Additional file 1: Table S1). Participants who dropped out or died had lower values in global health status/QoL, physical functioning, and role functioning at baseline than participants who completed the study (Additional file 1: Table S2).

### Characteristics associated with drop-out or death during follow-up

**Fig. 2:**
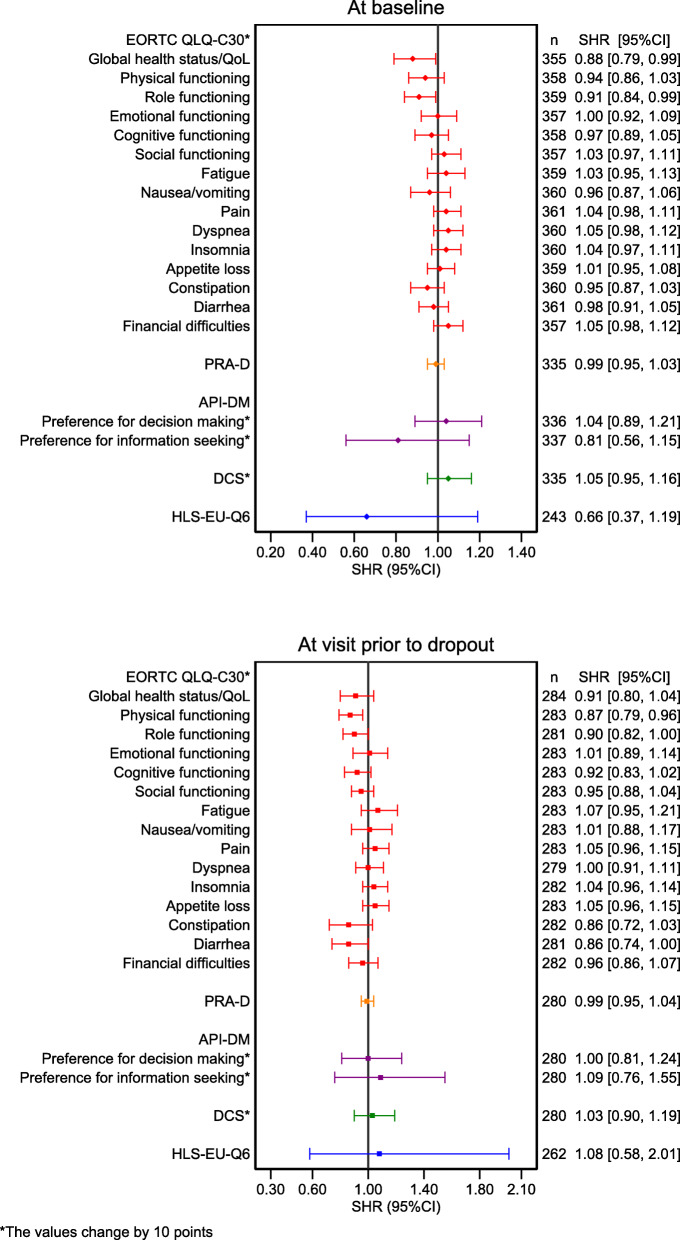
Patient-reported outcomes at baseline and at the visit prior to drop out, and their association to time-to-dropout, with death as a competing risk. *n* = the observation number, SHR = Sub-hazard ratio, CI=Confidence interval, High values of global health status/QoL and functional sub-scales in the EORTC QLQ-C30 indicate a better outcome, High values of symptom sub-scales (e.g fatigue, nausea/vomiting, pain etc.) and DCS indicate a poorer outcome

In our study, family status and poor subjective social support were related to drop-out (Table 1). Lower global health status/QoL and role functioning of the EORTC QLQ-C30 at baseline were associated with a higher risk of drop-out, as a difference in global health status/QoL and role functioning by 10 points resulted in a 12% (95%CI: 1–21%) and 9% (95%CI: 1–16%) higher risk of drop-out. In addition, low physical functioning of the EORTC QLQ-C30 at the visit before drop-out was associated with a higher likelihood of drop-out when considering death as a competing risk (Fig. 2).

**Table 1:** Participants’ demographic data as characteristics associated with time-to-dropout, with early death as a competing risk

	n	Time-to-dropout with death as a competing risk		Death	
		SHR	(95%CI)	HR	(95%CI)
**Study group**					
Intervention	150	1.23	(0.76, 1.99)	1.26	(0.84, 1.88)
Control	212	1		1	
**Age (years)**	362	1.01	(0.99, 1.03)	1.02	(1.00, 1.03)
**Study site**					
Study site 1	119	1			
Study site 2	98	1.77	(0.94, 3.33)	1.69	(0.99, 2.89)
Study site 3	145	1.60	(0.87, 2.93)	1.99	(1.22, 3.26)
**Sex**					
Male	219	1.35	(0.82, 2.24)	0.75	(0.51, 1.11)
Female	143	1		1	
**Family status**					
Married	226	1.15	(0.50, 2.67)	1.68	(0.84, 3.38)
Single	44	1		1	
Divorced/Widowed	63	2.71	(1.12, 6.56)	0.89	(0.36, 2.20)
**Time since diagnosis**					
≤ 6 months	187	1		1	
7–12 months	49	0.43	(0.17, 1.08)	1.06	(0.58, 1.93)
13–24 months	46	0.54	(0.22, 1.32)	1.53	(0.87, 2.67)
> 24 months	80	0.97	(0.54, 1.72)	1.33	(0.81, 2.19)
**Diagnosis**					
Acute leukemia	69	1		1	
Aggressive lymphoma	58	1.53	(0.64, 3.65)	0.55	(0.25, 1.23)
Malignant neoplasm of the bronchus and lungs	62	1.92	(0.84, 4.38)	1.78	(0.97, 3.27)
Metastatic colorectal cancer/colon carcinoma	78	1.39	(0.60, 3.21)	1.19	(0.64, 2.21)
Malignant neoplasm of the pancreas	32	1.94	(0.79, 4.77)	2.48	(1.27, 4.85)
Multiple myeloma and malignant plasma cell neoplasms	24	1.24	(0.40, 3.83)	0.56	(0.19, 1.65)
Metastasized malignant neoplasm of the breast	9	0.91	(0.11, 7.21)	0.41	(0.05, 3.10)
Others	30	2.13	(0.79, 5.74)	1.32	(0.58, 3.03)
**Education**					
Low	29	1.85	(0.90, 3.81)	0.92	(0.39, 2.15)
Medium	101	1.27	(0.72, 2.25)	1.58	(1.02, 2.45)
High	206	1		1	
**Social support (OSSS-3)**					
Poor (3–8)	35	2.10	(1.01, 4.35)	0.83	(0.39, 1.79)
Moderate (9–11)	158	1.32	(0.74, 2.36)	0.87	(0.56, 1.36)
Strong (12–14)	139	1		1	

*n* Number of observation, *SHR* Sub-hazard ratio, *HR* Hazard ratio, *CI* Confidence Interval

**Fig. 3:**
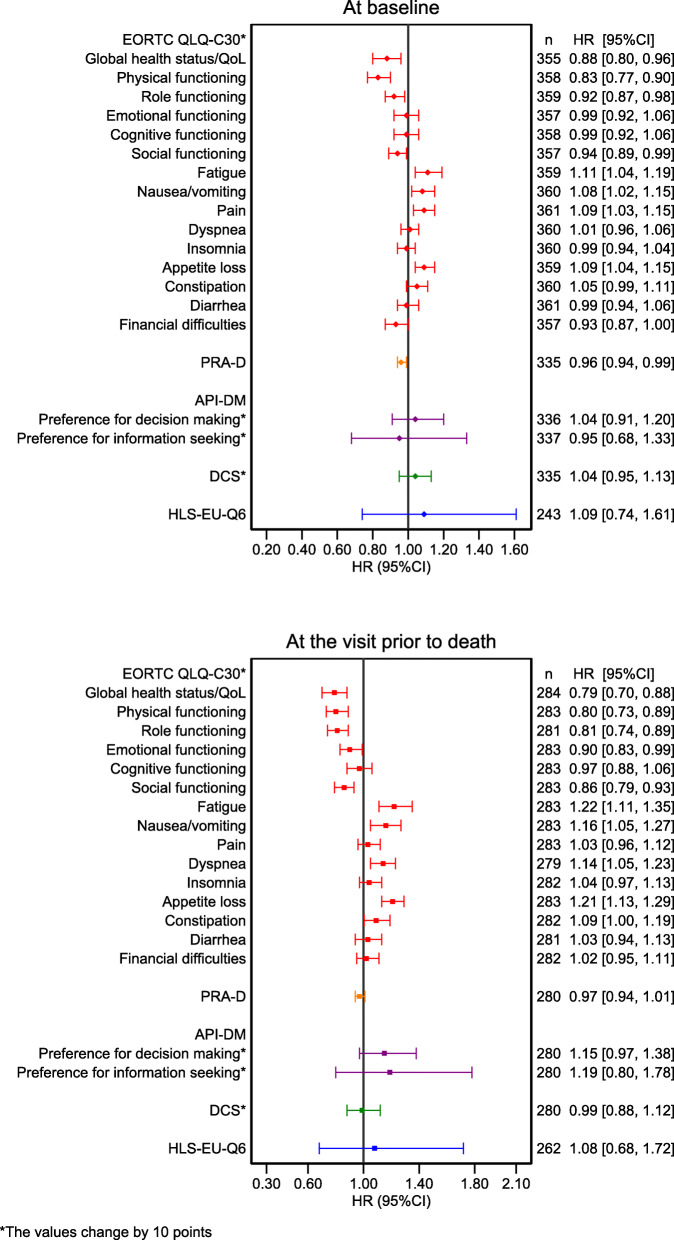
Patient-reported outcomes at baseline and at the visit prior to death, and their association to time-to-death. *n* = the observation number, HR = Hazard ratio, CI=Confidence interval, High values of global health status/QoL and functional sub-scales in the EORTC QLQ-C30 indicate a better outcome, High values of symptom sub-scales (e.g fatigue, nausea/vomiting, pain etc.) and DCS indicate a poorer outcome

Characteristics associated with shorter time to death were malignant neoplasm of the pancreas when compared to acute leukemia (HR = 2.48; 95%CI: 1.27–4.85), low levels of EORTC QLQ-C30 at baseline, low global health status/QoL, low physical functioning, low role functioning, and poorer outcomes on the symptom scale (fatigue, nausea and vomiting, dyspnea, and appetite loss) at the visit before death (Table 1 and Fig. 3). A poor doctor-patient relationship at baseline was also associated with a shorter time to death. There were also study site differences for time to death. Sensitivity analyses showed similar results, meaning that study sites, family status, and the EORTC QLQ-C30 were important factors for both drop-out and death (Additional file 1: Table S3–5).

**Fig. 4:**
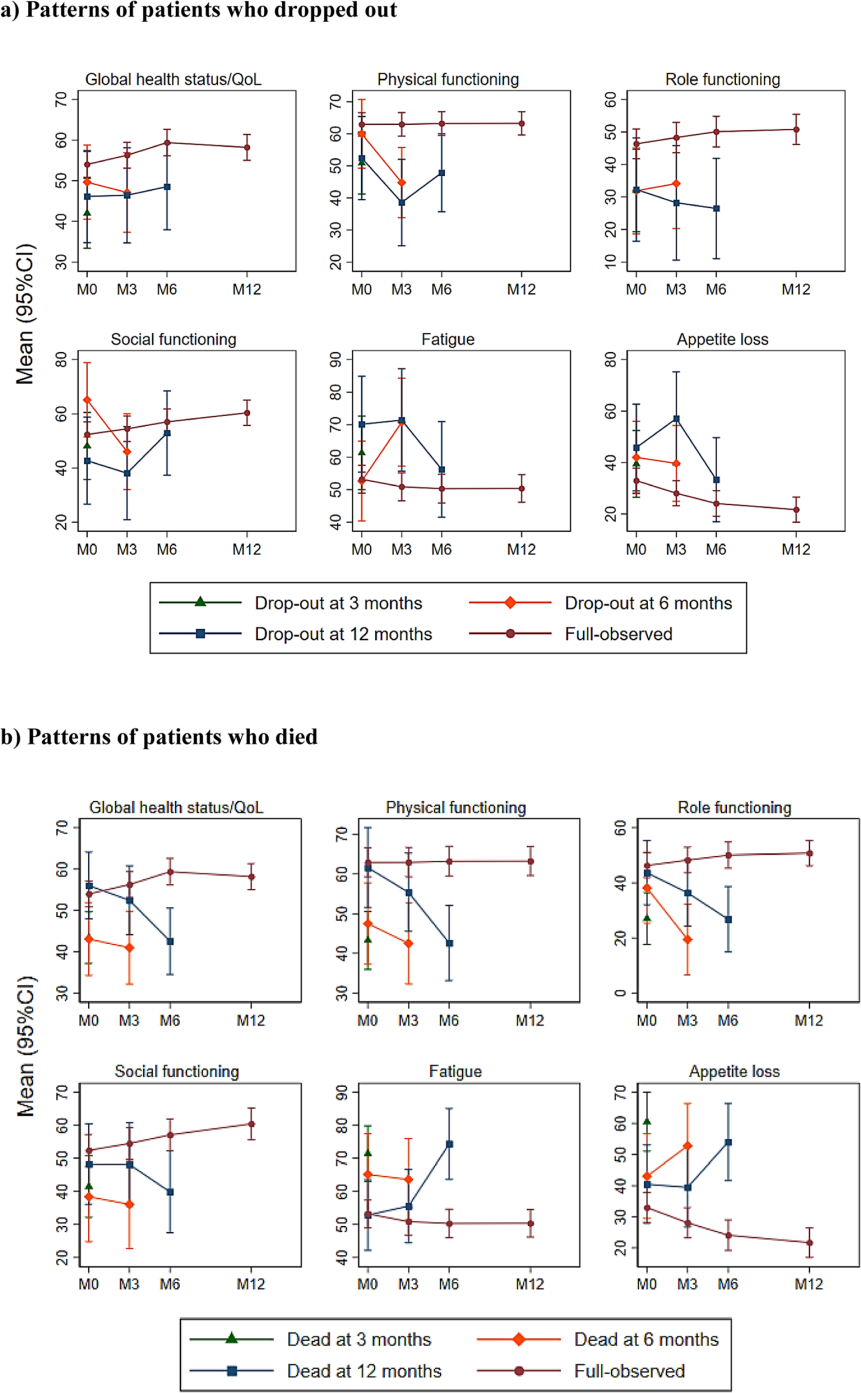
Patterns of patients who (**a**) dropped out or (**b**) died: Sub-scales of the EORTC QLQ-C30 versus follow-up times, stratified by time of drop-out or death. The possible range of the EORTC QLQ-C30 is 0–100, with higher values indicating a better QoL in global health status/QoL, physical functioning, role functioning, social functioning, and lower values indicating better symptoms for fatigue and appetite loss

The trajectories of the EORTC QLQ-C30 over time were substantially different for participants who dropped out, passed away during the study time, and those who completed the study visits (Fig. 4a and b). It is clear that the QoL, functionals and symptoms values decreased before the participant missed the visit due to death, whereas these values did not decrease in those who dropped out. However, patients who have low baseline values in global health status/QoL and poorer functionalities of the EORTC QLQ-C30 were more likely to drop out or die early.

## Discussion

The purpose of this study was to investigate the patient characteristics and PROs associated with drop-out and death in a non-randomized intervention study for severely ill cancer patients. Family status, subjective social support, low values of global health status/QoL and role functioning of the EORTC QLQ-C30 at baseline and low value of physical functioning at the visit before drop-out were associated with time-to-dropout, while the study site, diagnosis, low values of global health status/QoL, physical functioning and role functioning, strong symptoms (e.g. fatigue, nausea, vomiting or appetite loss) of the EORTC QLQ-C30 at both baseline and the visit before death were associated with time-to-death.

### Drop-out rate in the OSCAR study

Around 50% of the subjects completed all the study visits in OSCAR, meaning that there was a relatively high drop-out rate in our study when compared to prior oncological studies [6, 7, 9, 21, 22]. Surprisingly, there was no marked differential drop-out between intervention and control groups, despite our expectation of lower drop-out rates for the control group because the participants would want to benefit from intervention. We found slightly higher rates of drop-out and death in the intervention group than in the control group (Additional file 1: Figure S1). The main reason for not completing the study was premature death, accounting for approximately 60% (102/169) of patient discontinuation. However, the overall attrition (drop-out) rate of 18.5% in the OSCAR (withdrawal, 15.2%; loss-to-follow up, 2.8%; other reasons, 0.5%) was modest when compared to rates reported in other oncological studies, which ranged from 18 to 31% [6, 9, 23]. A high rate of premature death was expected in this population of severely affected cancer patients, in line with the study’s aim of including severely affected cancer patients.

### Patient characteristics associated with drop-out

Our findings show that family status (being divorced or widowed) and a lack of subjective social support were positively associated with early drop out. Similar findings were observed among cancer patients in a cluster-randomized controlled trial [9]. However, other studies could not confirm this association [6, 7]. Additionally, a lack of social support was associated with early drop-out among cancer patients. It has been reported that the family status plays a role as social support and has a positive effect on a patient’s health, quality of life, and coping behavior [24]. Therefore, a lack of social support from family or friends might be one explanation for a lack of desire to continue the study.

In our study, we did not find an association between general characteristics (e.g age, gender and education) and drop-out. Older age was not strongly related to drop-out or death in our study, although there was a weak association between older age and early death. This result is in line with previous cancer studies [6, 7, 21], though some studies have reported contrary findings [9, 10]. Males seemed more likely to drop out early in our study, though this association was reversed in some other studies [22, 23]. Other studies found no association between sex and the probability of dropping out [7, 9]. Participants with lower educational status dropped out early more often, but this association was weak in our study. Similarly, Spiers et al. and Roick et al. found that low education was associated with the lack of a follow-up [9, 10].

The probability of early death differed between study sites and could have been related to the difference in distribution of diagnosis between the study sites. We found that 70% of study site 3’s cancer patients had malignant neoplasm of the bronchus and lungs, metastatic colorectal cancer or colon carcinoma, or malignant neoplasm of the pancreas, while study site 1 enrolled around 15% of these cancer types and about 60% with acute leukemia or aggressive lymphoma (data not shown). It is relevant to our findings that patients who were diagnosed with malignant neoplasm of the pancreas had a higher risk of early death. The prognosis for pancreatic cancer is generally poor, meaning that the overall survival rate is low: a five-year survival rate of ca. 10% compared with patients with acute lymphocytic leukemia (72.1%) [25].

### Patient-reported outcomes associated with drop-out

Our results show that lower levels of PROs are in fact associated with both a higher probability of drop-out and premature death. Cancer patients with a poor quality of life and high symptom burden at baseline and at the visit before drop-out were at high risk of early drop-out and death in our study. These findings are similar to those of other studies [6, 7, 21, 22]. In addition to this, we found that participants who dropped out due to illness or other reasons had a lower disease burden and better functionalities than those who died during the study period. We found that fatigue, nausea, vomiting, and appetite loss were associated with early death for both time points at baseline and at the visit before drop-out. These symptoms have been identified as early signs of upcoming death in cancer patients, especially fatigue symptoms [26]. In other words, our results show that patients who died prematurely tend to show a trend of progressive deterioration, indicated by a reduction in their health-related quality of life scores and lower baseline scores, while the patients who completed the study were stable throughout.

### Finding application for further statistical analyses

Our findings, that baseline characteristics and QoL were associated with drop-out, may be useful for determining the potential missing data mechanism, which is a prerequisite to choosing how to handle missing PRO data, such as imputation methods. In addition to this, poor baseline QoL scores should encourage researchers to assess more auxiliary data, such as the Eastern Cooperative Oncology Group (ECOG) performance status. It should also encourage them to research the reasons for incomplete PROMs questionnaires being collected, assisting in determining what is missing and using this auxiliary data as a covariate in the model, or in multiple imputation methods [27]. In the relationship between symptom severity and missing data in health-related QoL due to drop-out or death, the missing data mechanism is clearly not completely random (MCAR). The pattern of PROs before dropping out and death may suggest that it is missing at random (MAR) or not missing at random (MNAR). The results suggest that, within the OSCAR study and similar studies, gaps in information have to be handled appropriately for a primary endpoint analysis to yield unbiased results. Complete case analysis or last observation carried forward (LOCF) methods would not be appropriate, especially in health-related QoL outcomes. However, yielding unbiased results depends on the appropriate handling of missing data within the analytical approach [28]. Alternative models (like pattern mixture models and joint models) might be used if the PROs data is MNAR [27, 29]. Independent of the particular statistical method used to handle missing PRO data, sensitivity analyses should be conducted and reported, regardless of the type of missing data mechanism [27].

### Implications for further studies with advanced cancer patients

Our results show that a high drop-out rate in advanced cancer patients is to be expected, especially when due to premature death. Rates of early death are associated with certain diagnosis groups, such as malignant neoplasm of the pancreas. Therefore, the adjustment of sample sizes should have an attrition rate of up to 50% when planning a study. Inclusion criteria should be more specific (such as estimated life expectancy or ECOG performance status). An estimated life expectancy of more than 3 months is commonly used in cancer clinical trials [30]; however, choosing the estimated life expectancy depends on the subject matter of the study and their benefit from receiving an intervention. However, recruitment when patients are admitting to the intensive care unit should be avoided to reduce drop-out due to in-hospital death. Otherwise, the study assistant should do a post-recruitment if the participant dies shortly after the initial recruitment. The researcher should actively monitor baseline characteristics, as the likelihood of dropping out depends on a patient’s social background (marriage, social support), gender, and educational status. Fatigue, nausea, vomiting, and appetite loss are signs of imminent death. Prompt changes in the follow-up phase study process might be necessary, such as close, continued monitoring after a patient misses an assessment and shorter time windows between follow-up visits and post-recruitment. Reasons for mis-measurement and discontinuation are very importance, especially when PROs are the primary research outcome. These reasons may help statisticians to handle the missing data and choose an appropriate statistical approach to the type of missing mechanism.

### Limitations

No information on the severity of a patient’s disease (such as the stage of cancer) which could affect the time until death and multivariable analyses were not possible, and the statistical power was restricted by multi-collinearity within the PROs and a low number of observations in some variables, such as education, family status, and HLS-EU-Q6. This exploratory study was done with no correction for multiple testing and without a multivariable analysis.

## Conclusions

Our exploratory study shows that participants with a low quality of life, poor symptoms and a lack of social support are more likely to discontinue a study earlier than patients with better values, resulting in a higher rate of missing patient-reported outcomes. This should be taken into account when planning a study of advanced cancer patients by monitoring in those who have a low baseline quality of life. Expected high mortality rates of up to 50% during the study time should already be considered in sample size calculations. Follow-up periods and study durations should be planned well. Continued monitoring is useful for characterizing the study sample and reacting quickly, thus avoiding a high drop-out rate. Methods of identifying the factors related to the drop-out similar to those presented here are useful for determining the missing data mechanism and informing choice of statistical methods for primary endpoint analysis. Methods for handling missing data might be applied appropriately, interpreting results of patient-reported outcomes should be done within caution, and the reasons for dropping out and how this may impact the findings should be discussed.

## Supplementary Information

**Additional file 1.**

## Data Availability

The datasets analyzed during the current study are not publicly available due to privacy and ethical concerns, and neither the data nor the source of the data can be made available. Upon request, the analysis code is available from the author.

## Abbreviations

API-DM: The German modified version of the Autonomy Preference Index
CASMIN: The Comparative Analysis of Social Mobility in Industrial Nations
CI: Confidence Interval
DCS: Decision Conflict Scale
EORTC QLQ-C30: The European Organization for Research and Treatment of Cancer’s Quality of Life Questionnaire
HL: Health Literacy
HLS-EU-Q6: The European Health Literacy Survey
HR: Hazard Ratio
IQR: Interquartile Range
LOCF: Last Observation Carried Forward
MAR: Missing at Random
MCAR: Missing Completely at Random
MNAR: Missing Not at Random
OSCAR: The Oncological Social Care Project
PRA-D: Patient Reaction Assessment
PROs: Patient-reported Outcomes
PROMs: Patient-reported Outcome Measures
OSSS-3: Oslo Social Support Scale
QoL: Quality of Life
SD: Standard Deviation
SHR: Sub-hazard Ratio
