# Supplementary material for: Characteristics and patient-reported outcomes associated with dropout in severely affected oncological patients: an exploratory study
Source: BMC Med Res Methodol. 2021 Apr 20;21:77. doi: 10.1186/s12874-021-01259-0 (PMC8059010; doi:10.1186/s12874-021-01259-0)
Supplement: Supplementary file 1 — Additional file 1. [file 12874_2021_1259_MOESM1_ESM.docx]

**Supplementary Materials**

**Characteristics and patient-reported outcomes associated with dropout in severely affected oncological patients: An exploratory study**

Pimrapat Gebert^1,2^, Daniel Schindel^3^, Johann Frick^3^, Liane Schenk*^3^and Ulrike Grittner*^1,2^

*These authors made equal contributions

^1^ Berlin Institute of Health at Charité –Universitätsmedizin Berlin, Charitéplatz 1, 10117 Berlin, Germany

^2^ Charité – Universitätsmedizin Berlin, corporate member of Freie Universität Berlin and Humboldt-Universität zu Berlin, Institute of Biometry and Clinical Epidemiology, Charitéplatz 1, 10117 Berlin, Germany

^3^ Charité – Universitätsmedizin Berlin, corporate member of Freie Universität Berlin and Humboldt-Universität zu Berlin, Institute of Medical Sociology and Rehabilitation Science, Charitéplatz 1, 10117 Berlin, Germany

**
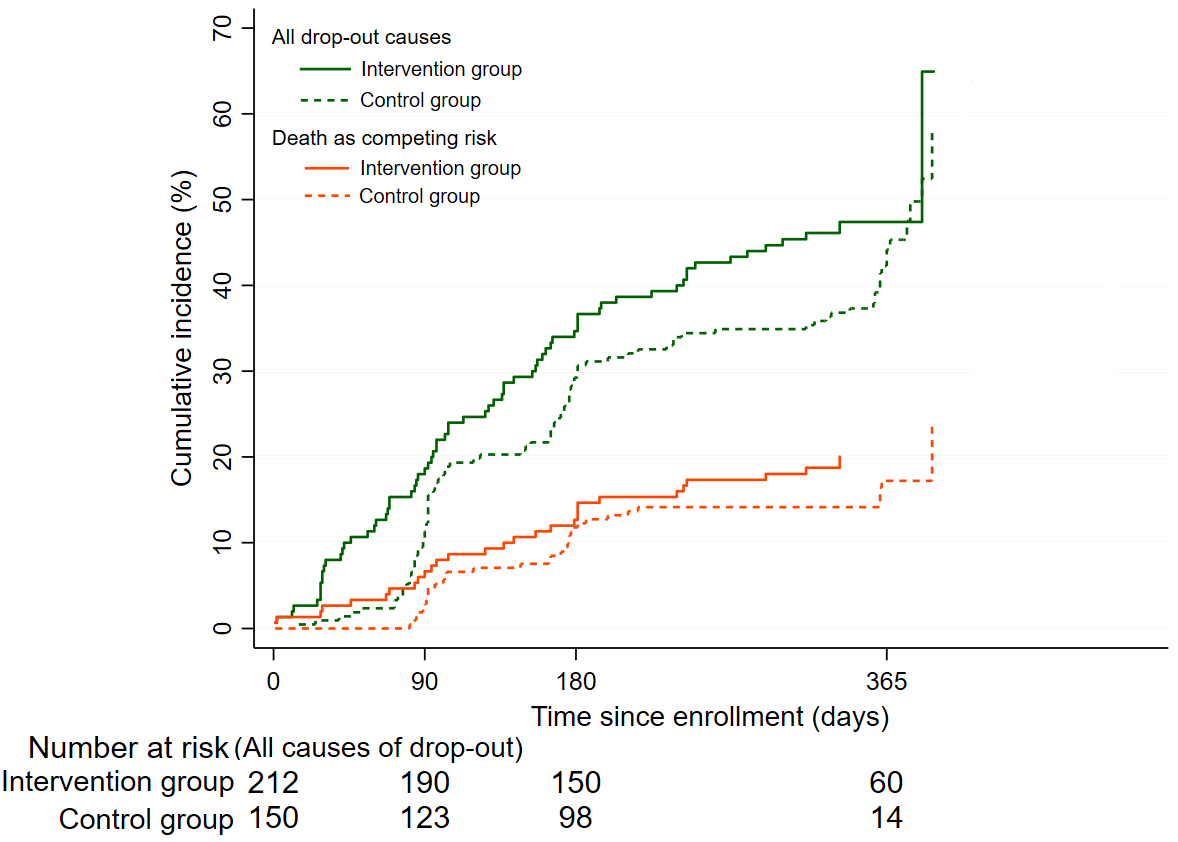
**

**Figure S1** Cumulative incidence of all drop-out causes (green line) and drop-out with death as competing risk (orange line) by intervention group (dash line) and control group (dot line)

**Table S1** Demographic data of the OSCAR study separately by type of participants

|  | **Total**  **n=362** | | **Participants with completed**  **the follow-up visits**  **n=193 (53.3%)** | | **Participants with discontinuous the study**  **n=169 (46.7%)** | | | |
| --- | --- | --- | --- | --- | --- | --- | --- | --- |
|  |  |  |  |  | **Drop-out**  **n=67 (18.5%)** | | **Death**  **n=102 (28.2%)** | |
| **Follow-up time (days)** |  | |  | |  | |  | |
| Median (IQR) | 333 (154, 361) | | 357 (339, 367) | | 164 (91, 199) | | 118 (77, 238) | |
| **Study group** |  | |  | |  | |  | |
| Intervention | 150 | (41.4%) | 79 | (40.9%) | 29 | (43.3%) | 42 | (41.2%) |
| Control | 212 | (58.6%) | 114 | (59.1%) | 38 | (56.7%) | 60 | (58.8%) |
| **Study site** |  |  |  |  |  |  |  |  |
| Study site 1 | 119 | (32.9%) | 78 | (40.4%) | 16 | (23.9%) | 25 | (24.5%) |
| Study site 2 | 98 | (27.1%) | 44 | (22.8%) | 23 | (34.3%) | 31 | (30.4%) |
| Study site 3 | 145 | (40.0%) | 71 | (36.8%) | 28 | (41.8%) | 46 | (45.1%) |
| **Age (years)** |  | |  | |  | |  | |
| Mean (SD, Min-Max) | 63 (13, 19 – 85) | | 62 (13, 19 – 85) | | 64 (14, 24 – 85) | | 65 (13, 25 – 85) | |
| **Sex** |  | |  | |  | |  | |
| Male | 219 | (60.5%) | 118 | (61.1%) | 45 | (67.2%) | 56 | (54.9%) |
| Female | 143 | (39.5%) | 75 | (38.9%) | 22 | (32.8%) | 46 | (45.1%) |
| **Family status** |  | |  | |  | |  | |
| Married | 226 | (62.4%) | 125 | (64.8%) | 33 | (49.3%) | 68 | (66.7%) |
| Single | 44 | (12.2%) | 29 | (15.0%) | 6 | (9.0%) | 9 | (8.8%) |
| Divorced/Widowed | 63 | (17.4%) | 33 | (17.1%) | 20 | (29.8%) | 10 | (9.8%) |
| Missing | 29 | (8.0%) | 6 | (3.1%) | 8 | (11.9%) | 15 | (14.7%) |
| **Time since diagnosis (months)** |  | |  | |  | |  | |
| Median (IQR) | 6 | (2, 22) | 6 | (2, 21) | 4 | (2, 24) | 9 | (2, 22) |
| ≤6 months | 187 | (51.7%) | 101 | (52.3%) | 40 | (59.7%) | 46 | (45.1%) |
| 7 – 12 months | 49 | (13.5%) | 30 | (15.5%) | 5 | (7.5%) | 14 | (13.7%) |
| 13 – 24 months | 46 | (12.7%) | 22 | (11.4%) | 6 | (9.0%) | 18 | (17.7%) |
| > 24 months | 80 | (22.1%) | 40 | (20.7%) | 16 | (23.9%) | 24 | (23.5%) |
| **Diagnosis (ICD-10-Code)** |  |  |  |  |  |  |  |  |
| Acute leukemia | 69 | (19.1%) | 41 | (21.2%) | 9 | (13.4%) | 19 | (18.6%) |
| Aggressive lymphoma | 58 | (16.0%) | 38 | (19.7%) | 11 | (16.4%) | 9 | (8.8%) |
| Malignant neoplasm of bronchus and lung | 62 | (17.1%) | 25 | (13.0%) | 14 | (20.9%) | 23 | (22.6%) |
| Metastatic colorectal cancer/colon carcinoma | 78 | (21.6%) | 43 | (22.3%) | 13 | (19.4%) | 22 | (21.6%) |
| Malignant neoplasm of pancreas | 32 | (8.8%) | 8 | (4.2%) | 8 | (11.9%) | 16 | (15.7%) |
| Multiple myeloma and malignant plasma cell neoplasms | 24 | (6.6%) | 16 | (8.3%) | 4 | (6.0%) | 4 | (3.9%) |
| Metastasized malignant neoplasm of breast | 9 | (2.5%) | 7 | (3.6%) | 1 | (1.5%) | 1 | (1.0%) |
| Others | 30 | (8.3%) | 15 | (7.8%) | 7 | (10.5%) | 8 | (7.8%) |
| **Education** |  | |  | |  | |  | |
| Low | 29 | (8.0%) | 15 | (7.8%) | 8 | (11.9%) | 6 | (5.9%) |
| Medium | 101 | (27.9%) | 49 | (25.4%) | 19 | (28.4%) | 33 | (32.4%) |
| High | 206 | (56.9%) | 123 | (63.7%) | 32 | (47.8%) | 51 | (50.0%) |
| Missing | 26 | (7.2%) | 6 | (3.1%) | 8 | (11.9%) | 12 | (11.8%) |
| **Social support (OSSS-3)** |  | |  | |  | |  | |
| Poor (3 – 8) | 35 | (9.7%) | 17 | (8.8%) | 10 | (14.9%) | 8 | (7.8%) |
| Moderate (9 – 11) | 158 | (43.7%) | 90 | (46.6%) | 29 | (43.3%) | 39 | (38.2%) |
| Strong (12 – 14) | 139 | (38.4%) | 81 | (42.0%) | 19 | (28.4%) | 39 | (38.2%) |
| Missing | 30 | (8.3%) | 5 | (2.6%) | 9 | (13.4%) | 16 | (15.7%) |

IQR=Interquartile range, SD=Standard deviation

**Table S2** Patient-reported outcomes at baseline

|  | **Total** | | **Participants with completed**  **the follow-up visits** | | **Participants with discontinuous**  **the study** | | | |
| --- | --- | --- | --- | --- | --- | --- | --- | --- |
|  |  |  |  |  | **Drop-out** | | **Death** | |
| **EORTC QLQ-C30** - Mean (SD) |  |  |  |  |  |  |  |  |
| Global health status/QoL | 50.6 | (21.9) | 54.3 | (21.6) | 45.7 | (22.8) | 46.9 | (21.9) |
| **Functional scales** |  |  |  |  |  |  |  |  |
| Physical functioning | 57.6 | (25.4) | 63.1 | (24.3) | 53.9 | (25.0) | 49.6 | (25.4) |
| Role functioning | 40.6 | (33.8) | 46.6 | (33.9) | 31.5 | (32.2) | 35.0 | (32.5) |
| Emotional functioning | 57.2 | (27.1) | 57.7 | (26.8) | 56.8 | (27.1) | 56.4 | (28.0) |
| Cognitive functioning | 73.9 | (29.1) | 74.5 | (27.1) | 71.7 | (31.5) | 74.3 | (31.3) |
| Social functioning | 49.7 | (35.1) | 52.2 | (33.1) | 53.3 | (35.7) | 42.7 | (37.7) |
| **Symptom scales/items** |  |  |  |  |  |  |  |  |
| Fatigue | 57.7 | (28.9) | 53.4 | (28.8) | 60.4 | (29.5) | 64.1 | (27.6) |
| Nausea and vomiting | 17.4 | (26.7) | 14.9 | (24.8) | 15.4 | (26.8) | 23.4 | (29.3) |
| Pain | 37.3 | (36.1) | 31.7 | (33.9) | 41.9 | (36.9) | 44.8 | (38.2) |
| Dyspnea | 39.4 | (38.3) | 37.3 | (37.0) | 45.1 | (41.8) | 39.5 | (38.3) |
| Insomnia | 43.9 | (38.9) | 43.4 | (38.2) | 48.7 | (42.9) | 41.8 | (37.7) |
| Appetite loss | 39.6 | (39.1) | 33.0 | (38.1) | 41.9 | (41.1) | 50.8 | (37.3) |
| Constipation | 21.9 | (33.8) | 21.2 | (32.3) | 16.9 | (31.8) | 26.1 | (37.4) |
| Diarrhea | 21.9 | (32.7) | 22.5 | (32.7) | 20.2 | (32.5) | 21.9 | (33.0) |
| Financial difficulties | 17.9 | (30.4) | 18.3 | (31.1) | 23.1 | (34.8) | 13.9 | (25.5) |
| **PRA-D** - Mean (SD) | 29.8 | (6.5) | 30.7 | (5.5) | 29.2 | (7.8) | 28.2 | (7.1) |
| **API-DM** - Mean (SD) |  |  |  |  |  |  |  |  |
| Preference for decision making | 52.8 | (14.9) | 52.2 | (15.3) | 53.6 | (13.6) | 53.7 | (14.9) |
| Preference for information seeking | 96.1 | (6.5) | 96.4 | (5.5) | 95.2 | (8.2) | 96.1 | (7.3) |
| **DCS -** Median (IQR) | 15 | (0, 40) | 15 | (0, 35) | 18 | (0, 45) | 15 | (0, 43) |
| **HLS-EU-Q6** |  |  |  |  |  |  |  |  |
| Mean (SD) | 2.76 | (0.66) | 2.78 | (0.61) | 2.60 | (0.83) | 2.80 | (0.65) |
| [1-2]=inadequate HL | 36 | (9.9%) | 18 | (9.3%) | 10 | (14.9%) | 8 | (7.8%) |
| (2-3)=problematic HL | 116 | (32.0%) | 66 | (34.2%) | 19 | (28.4%) | 31 | (30.4%) |
| [3-4]=sufficient HL | 91 | (25.1%) | 57 | (29.5%) | 12 | (17.9%) | 22 | (21.6%) |
| Missing | 119 | (32.9%) | 52 | (26.9%) | 26 | (38.8%) | 41 | (40.2%) |

HL=Health literacy, IQR=Interquartile range, SD=Standard deviation

**Table S3** Participants’ demographic data as a predictor of drop-out with and without death-related using Multinomial logistic regression

|  | **Drop-out** | | **Death** | |
| --- | --- | --- | --- | --- |
|  | **RR** | **(95%CI)** | **RR** | **(95%CI)** |
| **Study group** |  |  |  |  |
| Intervention | 1.10 | (0.63, 1.93) | 1.01 | (0.62, 1.64) |
| Control | 1 |  | 1 |  |
| **Age (years)** | 1.01 | (0.99, 1.04) | 1.02 | (1.00, 1.04) |
| **Study site** |  |  |  |  |
| Study site 1 | 1 |  | 1 |  |
| Study site 2 | 2.55 | (1.22, 5.33) | 2.20 | (1.15, 4.18) |
| Study site 3 | 1.92 | (0.96, 3.85) | 2.02 | (1.13, 3.62) |
| **Sex** |  |  |  |  |
| Male | 1.30 | (0.72, 2.34) | 0.77 | (0.48, 1.26) |
| Female | 1 |  | 1 |  |
| **Family status** |  |  |  |  |
| Married | 1.28 | (0.49, 3.33) | 1.75 | (0.78, 3.92) |
| Single | 1 |  | 1 |  |
| Divorced/Widowed | 2.93 | (1.04, 8.29) | 0.98 | (0.35, 2.73) |
| **Time since diagnosis** |  |  |  |  |
| ≤6 months | 1 |  | 1 |  |
| 7 – 12 months | 0.42 | (0.15, 1.16) | 1.02 | (0.50, 2.11) |
| 13 – 24 months | 0.69 | (0.26, 1.82) | 1.80 | (0.88, 3.67) |
| > 24 months | 1.01 | (0.51, 2.01) | 1.32 | (0.71, 2.44) |
| **Diagnosis** |  |  |  |  |
| Acute leukemia | 1 |  | 1 |  |
| Aggressive lymphoma | 1.32 | (0.50, 3.53) | 0.51 | (0.21, 1.27) |
| Malignant neoplasm of bronchus and lung | 2.55 | (0.96, 6.76) | 1.99 | (0.91, 4.35) |
| Metastatic colorectal cancer/colon carcinoma | 1.38 | (0.53, 3.57) | 1.10 | (0.52, 2.33) |
| Malignant neoplasm of pancreas | 4.56 | (1.35, 15.38) | 4.32 | (1.57, 11.83) |
| Multiple myeloma and malignant plasma cell neoplasms | 1.14 | (0.31, 4.23) | 0.54 | (0.16, 1.83) |
| Metastasized malignant neoplasm of breast | 0.65 | (0.07, 5.97) | 0.31 | (0.04, 2.69) |
| Others | 2.13 | (0.67, 6.72) | 1.15 | (0.42, 3.18) |
| **Education** |  |  |  |  |
| Low | 2.05 | (0.80, 5.26) | 0.96 | (0.35, 2.63) |
| Medium | 1.49 | (0.77, 2.88) | 1.62 | (0.94, 2.81) |
| High | 1 |  | 1 |  |
| **Social support (OSSS-3)** |  |  |  |  |
| Poor (3 – 8) | 2.51 | (0.99, 6.34) | 0.98 | (0.39, 2.46) |
| Moderate (9 – 11) | 1.37 | (0.72, 2.64) | 0.90 | (0.53, 1.54) |
| Strong (12 – 14) | 1 |  | 1 |  |

RR=Relative risk ratio, CI=Confidence interval

**Table S4** Patient-reported outcomes at baseline (t0) as a predictor of drop-out with and without death-related using Multinomial logistic regression

|  | **Drop-out** | | **Death** | |
| --- | --- | --- | --- | --- |
|  | **RR** | **(95%CI)** | **RR** | **(95%CI)** |
| **EORTC QLQ-C30*** |  |  |  |  |
| Global health status/QoL | 0.83 | (0.73, 0.95) | 0.85 | (0.76, 0.96) |
| **Functional scales** |  |  |  |  |
| Physical functioning | 0.86 | (0.77, 0.97) | 0.80 | (0.73, 0.89) |
| Role functioning | 0.87 | (0.80, 0.95) | 0.90 | (0.84, 0.97) |
| Emotional functioning | 0.99 | (0.89, 1.10) | 0.98 | (0.90, 1.07) |
| Cognitive functioning | 0.97 | (0.88, 1.06) | 1.00 | (0.92, 1.08) |
| Social functioning | 1.00 | (0.93, 1.09) | 0.93 | (0.86, 0.99) |
| **Symptom scales/items** |  |  |  |  |
| Fatigue | 1.09 | (0.99, 1.20) | 1.14 | (1.05, 1.24) |
| Nausea and vomiting | 1.01 | (0.90, 1.13) | 1.12 | (1.03, 1.22) |
| Pain | 1.08 | (1.00, 1.17) | 1.11 | (1.03, 1.18) |
| Dyspnea | 1.05 | (0.98, 1.13) | 1.02 | (0.95, 1.08) |
| Insomnia | 1.04 | (0.96, 1.11) | 0.99 | (0.93, 1.05) |
| Appetite loss | 1.06 | (0.99, 1.14) | 1.12 | (1.06, 1.20) |
| Constipation | 0.96 | (0.87, 1.05) | 1.04 | (0.97, 1.12) |
| Diarrhea | 0.98 | (0.90, 1.07) | 0.99 | (0.92, 1.07) |
| Financial difficulties | 1.05 | (0.96, 1.14) | 0.95 | (0.87, 1.03) |
| **PRA-D** | 0.96 | (0.92, 1.01) | 0.94 | (0.91, 0.98) |
| **API-DM*** |  |  |  |  |
| Preference for decision making | 1.07 | (0.88, 1.29) | 1.07 | (0.90, 1.26) |
| Preference for information seeking | 0.78 | (0.52, 1.18) | 0.92 | (0.62, 1.36) |
| **DCS*** | 1.07 | (0.96, 1.20) | 1.05 | (0.95, 1.16) |
| **HLS-EU-Q6** | 0.66 | (0.38, 1.12) | 1.04 | (0.66, 1.63) |
| [1-2]=inadequate HL | 2.64 | (0.98, 7.12) | 1.15 | (0.44, 3.03) |
| (2-3)=problematic HL | 1.37 | (0.61, 3.06) | 1.22 | (0.63, 2.33) |
| [3-4]=sufficient HL | 1 |  | 1 |  |

*The values change by 10 points

HL=Health literacy, RR= Relative risk ratio, CI=Confidence Interval

**Table S5** Patient-reported outcomes at the visit before drop-out as a predictor of drop-out with and without death-related using Multinomial logistic regression

|  | **Drop-out** | | **Death** | |
| --- | --- | --- | --- | --- |
|  | **RR** | **(95%CI)** | **RR** | **(95%CI)** |
| **EORTC QLQ-C30*** |  |  |  |  |
| Global health status/QoL | 0.83 | (0.72, 0.97) | 0.74 | (0.64, 0.85) |
| **Functional scales** |  |  |  |  |
| Physical functioning | 0.80 | (0.70, 0.91) | 0.76 | (0.68, 0.86) |
| Role functioning | 0.83 | (0.74, 0.93) | 0.77 | (0.69, 0.85) |
| Emotional functioning | 0.98 | (0.86, 1.11) | 0.88 | (0.79, 0.98) |
| Cognitive functioning | 0.90 | (0.80, 1.02) | 0.95 | (0.85, 1.06) |
| Social functioning | 0.90 | (0.81, 1.00) | 0.82 | (0.74, 0.90) |
| **Symptom scales/items** |  |  |  |  |
| Fatigue | 1.14 | (1.01, 1.28) | 1.28 | (1.14, 1.43) |
| Nausea and vomiting | 1.08 | (0.93, 1.27) | 1.20 | (1.07, 1.36) |
| Pain | 1.08 | (0.97, 1.19) | 1.05 | (0.96, 1.15) |
| Dyspnea | 1.05 | (0.95, 1.16) | 1.17 | (1.07, 1.28) |
| Insomnia | 1.07 | (0.97, 1.18) | 1.07 | (0.99, 1.17) |
| Appetite loss | 1.14 | (1.03, 1.26) | 1.28 | (1.17, 1.40) |
| Constipation | 0.87 | (0.73, 1.04) | 1.09 | (0.99, 1.20) |
| Diarrhea | 0.87 | (0.73, 1.03) | 1.03 | (0.93, 1.15) |
| Financial difficulties | 0.98 | (0.88, 1.10) | 1.04 | (0.96, 1.14) |
| **PRA-D** | 0.99 | (0.94, 1.04) | 0.97 | (0.93, 1.01) |
| **API-DM*** |  |  |  |  |
| Preference for decision making | 1.09 | (0.85, 1.40) | 1.24 | (1.00, 1.53) |
| Preference for information seeking | 1.09 | (0.68, 1.75) | 1.15 | (0.76, 1.76) |
| **DCS*** | 1.03 | (0.88, 1.19) | 0.99 | (0.87, 1.13) |
| **HLS-EU-Q6** | 1.08 | (0.59, 1.98) | 1.09 | (0.64, 1.86) |
| [1-2]=inadequate HL | 0.98 | (0.34, 2.87) | 0.98 | (0.31, 3.13) |
| (2-3)=problematic HL | 0.76 | (0.33, 1.75) | 1.88 | (0.86, 4.14) |
| [3-4]=sufficient HL | 1 |  | 1 |  |

*The values change by 10 points

HL=Health literacy, RR= Relative risk ratio, CI=Confidence Interval
